# Comparison of Uric Acid Quantity with Different Food in Human Urine by Flow Injection Chemiluminescence Analysis

**DOI:** 10.1155/2013/854041

**Published:** 2013-10-22

**Authors:** Jiajia Wang, Xijuan Tan, Zhenghua Song

**Affiliations:** Key Laboratory of Synthetic and Natural Functional Molecule, Chemistry of Ministry of Education, College of Chemistry and Materials Science, Northwest University, Xi'an 710069, China

## Abstract

Based on the inhibitory effect of uric acid (UA) on luminol-Co^2+^ chemiluminescence (CL) system, a sensitive method for the determination of UA at nanomolar level by flow injection (FI) CL was proposed. The proposed method was successfully applied to real-time monitoring of UA excretion in human 24 h urine with different food intake, showing that meats, vegetables, and porridge intake caused differential UA excretions of 879, 798, and 742 mg, respectively. It was also found that UA concentrations in urine under the three kinds of food intake simultaneously reached maximum at 2 h after meals with the values of 417, 318, and 288 *μ*g mL^−1^, respectively. The UA concentration in human serum was also determined by this approach, and the possible mechanism of luminol-Co^2+^-UA CL reaction was discussed in detail.

## 1. Introduction

**Figure 1:**
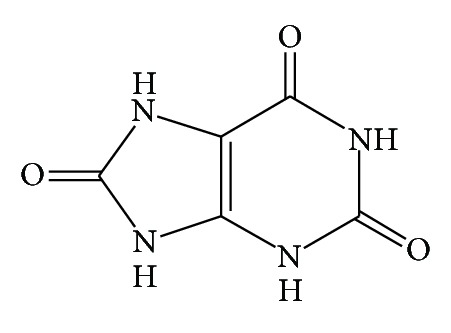
Chemical structure of UA.

Uric acid (2,6,8-trihydroxypurine, UA, Figure 1), the principal breakdown product of human purine metabolism, is mainly excreted by the kidney [1]. Normally, most of UA produced from the catabolism is reabsorbed into the blood circulation system and then passes through the kidney into urine. When UA in human urine or serum exceeds the normal physiological range, it may cause gout [2], kidney injury [3], metabolic syndrome [4], cardiovascular disease [5], and type II diabetes [6]. Consequently, monitoring the concentration of UA in bodily fluids is essential in diagnosis and treatment of the corresponding diseases. Many methods have been reported for the determination of UA, such as capillary electrophoresis (CE) [7], enzymatic assay (EA) [8], high-performance liquid chromatography (HPLC) [9, 10], differential pulse voltammetric (DPV) [11, 12], and UV-Vis spectroscopy [13, 14]. Chemiluminescence (CL) has also been developed for UA determination [15–17].

Luminol with Co^2+^ reaction is known as a classic CL system [18]. The CL mechanism of Co^2+^ remarkably enhancing luminol intensity has been well studied [19, 20], and a variety of applications with luminol-Co^2+^ CL system in trace analysis [21, 22] have been reported. For example, it has been applied to the determination of hydrogen peroxide [23, 24], acetaminophen [25], lanthanides [26], carbaryl [27], captopril [28], vitamin B_12_ [29], gibberellic acid [30], and radical scavenging potential of ascorbic acid [31]. With luminol-H_2_O_2_-Co^2+^ CL system, the determination of UA have been reported [32], while no real-time analysis of urine UA excretion with luminol-Co^2+^ CL system has been described so far.

 Flow injection (FI) analysis combined with CL, FI-CL, which possess merits of low limit of detection (LOD), fast response time, less reagent consumption, and simple instrumentation as well as automation, has become a very useful analytical tool in different scientific fields from food safety [33–35] to environmental chemistry [36–38]. In this work, it was found that UA could obviously quench the CL intensity of luminol-Co^2+^ in a flow system, with the decrements in CL intensity linearly proportional to the logarithm of UA concentrations ranging from 3.0 to 500.0 nmol L^−1^, giving the LOD of 1.0 nmol L^−1^ (3*σ*). The relative standard deviation (RSD) was 2.5% for determining 10.0* *nmol L^−1^ UA. At a flow rate of 2.0 mL min^−1^, a single UA determination cycle could be accomplished within 36 s, offering a sample throughput (ST) of 100 h^−1^. UA excretion in urine is known to be closely associated with food intake, and the normal excretion of UA is 250–740 *μ*g mL^−1^ per day [39]. Herein, quantitative monitoring of urine UA excretion with different food intake by FI-CL was proposed for the first time.

## 2. Experimental

### 2.1. Reagents

All chemical reagents in this work were of analytical reagent grade. Doubly deionized water purified in a Milli-Q system (Millipore, Bedford, MA, USA) with the resistivity of 18.2 MΩ cm was used for the preparation of solutions in the whole procedure. Luminol (Fluka, Biochemika, Switzerland) and UA (Sigma) were obtained from Xi'an Medicine Purchasing and Supply Station, China.

The luminol stock standard solution of 2.5 × 10^−2^ mol L^−1^ was prepared in 1.0 × 10^−1^ mol L^−1^ NaOH solution and kept in the dark. The Co^2+^ stock standard solution of 1.0 × 10^−3^ mol L^−1^ was prepared by dissolving 11.90 mg of CoCl_2_·6H_2_O (99%) in 50 mL calibrated flask with doubly deionized water. The UA stock standard solution of 1.0 × 10^−3^ mol L^−1^ was prepared in 1.0 × 10^−2^ mol L^−1^ NaOH solution and stored at 4°C. All the daily used working standard solutions were freshly prepared from the above stock standard solutions by appropriate dilution as required.

### 2.2. Apparatus

**Figure 2:**
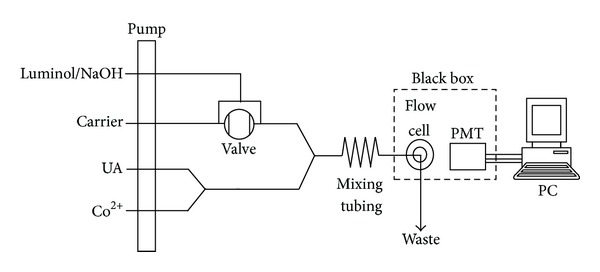
Schematic diagram for the determination of UA.

The schematic profile of equipment in the flow system (Xi'an Remax Analysis Instrument Co. Ltd., Xi'an, China) was depicted in Figure 2. The apparatus contained a sampling system (IFFM-E), a CL detector (IFFS-A), and a recorder (a computer with IFFM-E client system). The sampling system used a peristaltic pump with four channels to pump the reagents and a six-way valve with a loop of 100 *μ*L to quantitatively inject luminol. The CL detector, which was placed inside a black box for precluding the interference from ambient light, contained a flow cell and a photomultiplier tube (PMT). The flow cell, tightly positioned on the window of PMT, was a colorless glass tube (15 cm in length, 1.0 mm i.d.) with a spiral disk shape (2.0 cm i.d.). The CL signal from the flow cell was directly detected by the PMT without any wavelength discrimination and the output was recorded by the computer. The polytetrafluoroethylene (PTFE) tubing (1.0 mm i.d.) was used to deliver the reagents.

### 2.3. General Procedures

As shown in Figure 2, flow lines were inserted into luminol, carrier (doubly deionized water), UA, and Co^2+^ solutions, respectively. The pump was started with the stream of doubly deionized water at a constant flow rate of 2.0 mL min^−1^ to wash the whole system. Then 100 *μ*L of luminol solution was injected into the flow system by the six-way valve and, thereafter, merged with the premixed, homogeneous stream of UA and Co^2+^. The entire mixed solution was then delivered into the CL cell, producing CL emission. The CL signal was detected by PMT, and the PMT negative voltage was set as 700 V. The CL intensity decrement (Δ*I*_CL_ = *I*_0_ − *I*_*s*_), where *I*_0_ and *I*_*s*_ were defined as CL intensity in the absence and presence of UA, respectively, was used as the quantitative parameter for UA concentration.

### 2.4. Sample Preparation

The urine samples, which came from volunteers who have prehydrated, were collected in beakers with volumes recorded. The UA concentrations in urine samples were measured directly after an appropriate dilution. When necessary, urine samples were refrigerated at 4°C and were determined as soon as possible to guarantee the sample integrity.

The human serum samples were obtained from the Hospital of Northwest University. After centrifugation, 0.1 mL supernatant was diluted with doubly deionized water and mixed thoroughly for CL determination after dilution.

## 3. Results and Discussion

### 3.1. Optimum Experimental Conditions for the Determination of UA

 Two manifolds of quantitatively injecting luminol or UA sample into the carrier stream by six-way valve were investigated. It was found that two significantly different results were obtained. Using the manifold of quantitatively injecting luminol by six-way valve, a whole analytical process including sampling and washing could be accomplished within 36 s, offering a ST of 100 h^−1^ accordingly. Whereas by using the manifold of injecting UA, the same analytical process needed 120 s, yielding the ST of 30 h^−1^. It was clear that the ST of the manifold of injecting luminol shown in Figure 2 is about 3-fold to that of injecting UA. Therefore, the manifold depicted in Figure 2 was chosen in subsequent work.

**Figure 3:**
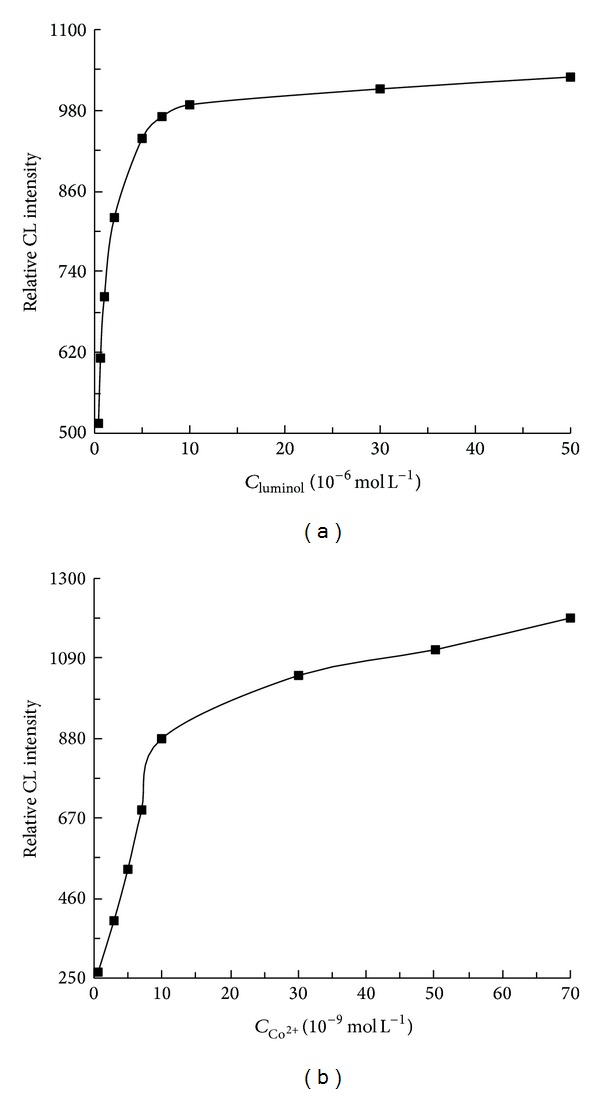
Effect of luminol (a) and Co^2+^ (b) concentrations on the CL intensity. (a) The concentration of Co^2+^ was 1.0 × 10^−8^ mol L^−1^; (b) the concentration of luminol was 5.0 × 10^−6^ mol L^−1^.

The effect of luminol concentration (5.0 × 10^−7^~5.0 × 10^−5^ mol L^−1^) and Co^2+^ concentration (7.0 × 10^−10^~7.0 × 10^−8^ mol L^−1^) on the CL intensity was investigated, respectively. As shown in Figure 3(a), it can be seen that the CL signal increased steadily with increasing concentration of luminol up to 5.0 × 10^−6^ mol L^−1^, above which the CL signal tended to be stable. Thus, 5.0 × 10^−6^ mol L^−1^ luminol was chosen as the optimal. It is also clear in Figure 3(b) that the CL intensity rose drastically when the Co^2+^ concentration increased to 1.0 × 10^−8^ mol L^−1^ and then increased slowly from a higher concentration. Therefore, 1.0 × 10^−8^ mol L^−1^ was chosen as the optimum concentration of Co^2+^. Owing to the nature of luminol CL reaction, which was favorable in alkaline medium, NaOH was added into luminol solution to increase the sensitivity of this CL system. A series of NaOH solutions ranging from 1.0 × 10^−4^ to 1.0 × 10^−1^ mol L^−1^ was tested. At 2.5 × 10^−2^ mol L^−1^ NaOH, the CL signal could reach a maximum value. Hence, 2.5 × 10^−2^ mol L^−1^ of NaOH was the optimal concentration for this CL system. 

The flow rate and mixing tube length had a great effect on the CL intensity. A lower flow rate would slow down the sampling rate and cause dilution effect. Although higher flow rate can increase signal to noise (S/N), the precision of this method decreased. Considering a good sensitivity, a better precision, and a lower reagent consumption, a flow rate of 2.0 mL min^−1^ was selected for this CL system. The effect of mixing tubing on CL intensity was also tested with the mixing tube lengths ranging from 5.0 to 20.0 cm. It was found that a 10.0 cm of mixing tube afforded the best results with good sensitivity and reproducibility. Accordingly, this length was chosen in the subsequent experiments.

### 3.2. The Relative CL Intensity-Time Profile

**Figure 4:**
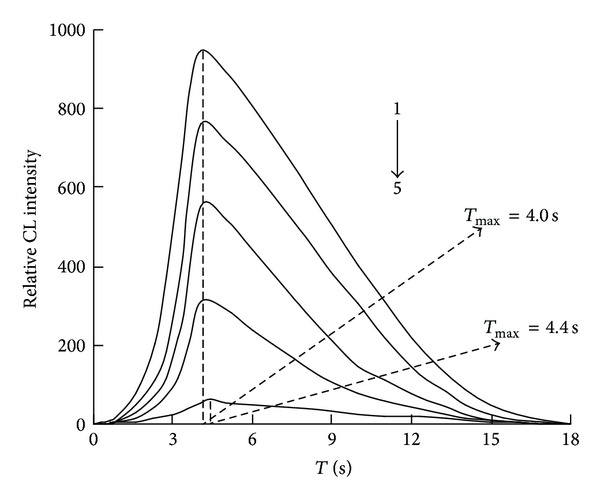
The relative CL intensity-time profile. Curve 1: luminol-Co^2+^ system; Curve 2–4: luminol-Co^2+^-UA system; Curve 5: luminol-dissolved oxygen system; Luminol: 5.0 × 10^−6^ mol L^−1^; Co^2+^: 1.0 × 10^−8^ mol L^−1^; UA: 5.0, 30.0 and 300.0 nmol L^−1^ for Curves 2, 3, and 4, respectively.

The relative CL intensity-time profiles of different reactions were given in Figure 4. It can be seen that the time (*T*_max⁡_) for reaching maximum CL intensity (*I*_max⁡_) of luminol-Co^2+^ reaction (curve 1) and luminol-dissolved oxygen reaction (curve 5) were 4.0 and 4.4 s with the *I*_max⁡_ of 939 and 59, respectively; the *T*_max⁡_ for luminol-Co^2+^ reaction in the presence of UA (curves 2–4) was 4.0 s, while the *I*_max⁡_ decreased from 939 to 750 (by 20.1%), 550 (by 41.4%), and 310 (by 66.9%) at 5.0, 30.0, and 300.0 nmol L^−1^ UA, correspondingly. These experiments were carried out by the optimized concentrations of luminol and Co^2+^, which were 5.0 × 10^−6^ mol L^−1^ and 1.0 × 10^−8^ mol L^−1^. 

### 3.3. Operational Stability of the FI-CL System

100 *μ*L of luminol solution (5.0 × 10^−6^ mol L^−1^) was injected into the carrier stream and thereafter merged with the premixing stream of Co^2+^ (1.0 × 10^−8^ mol L^−1^) and UA (10.0 and 100.0 nmol L^−1^, resp.). The relative CL intensity was recorded to test the stability and reproducibility of the luminol-Co^2+^ system. The FI system was regularly used over 8 h per day and the experiment lasted for 5 days. The results were listed in Table 1. Each result was the average of five separate determinations, and the RSDs were less than 3.5%. It suggested that the system exerted good stability.

### 3.4. Analytical Performance of UA Measurement

Under the optimal experimental conditions, a series of UA standard solutions was determined. It was found that the CL intensity of luminol-Co^2+^ system was obviously inhibited, and the decrements of CL intensity were linear with the logarithm of UA concentrations in the range of 3.0–500.0 nmol L^−1^, giving the calibration equation of Δ*I*_CL_ = 106.8ln⁡*C*_UA_ + 33.1 (*R* = 0.9982) with the LOD of 1.0 nmol L^−1^ (3*σ*). The RSDs (*n* = 5) at 5.0, 50.0, and 300.0 nmol L^−1^ UA were 2.9%, 2.3%, and 1.9%, respectively. At a flow rate of 2.0 mL min^−1^, a complete analytical process including sampling and washing could be accomplished within 36 s, offering a ST of 100 h^−1^. 

### 3.5. Interference Studies

To test for the interference of foreign substances on UA quantification, a potential interfering analyte was added in increasing amounts to the UA standard solution, and the relative CL intensity was examined with a relative error controlled at 5% level. The tolerable concentration ratios of foreign substances with respect to 10 ng mL^−1^ UA were over 4.0 × 10^4^ for SO_4_^2−^, NO_3_^−^, CO_3_^2−^, oxalate, PO_4_^3−^, glucose, and ethanol; 1.0 × 10^4^ for Ba^2+^, Zn^2+^ and HCO_3_^−^; 9.0 × 10^3^ for urea; 5.0 × 10^3^ for Mg^2+^ and Ca^2+^; 5.0 × 10^2^ for Ni^2+^ and Cr^3+^; 4.0 × 10^2^ for Cu^2+^, Mn^2+^, and ascorbic acid; 1.0 × 10^2^ for creatine and Fe^3+^/Fe^2+^; 20 for vitamin B_12_, respectively. Compounds abundant in human serum and urine such as lipids, proteins, and salts had no obvious interference for the determination of UA.

### 3.6. Possible Quenching Mechanism of UA on Luminol-Co^2+^ CL Reaction

 From Figure 4, it was clear that the *I*_max⁡_ of luminol-dissolved oxygen reaction in the presence of Co^2+^ increased from 59 to 939 and the *T*_max⁡_ changed from 4.4 to 4.0 s; it also can be seen that the *I*_max⁡_ of luminol-Co^2+^ system in the presence of UA decreased obviously at increasing UA concentration with the identical *T*_max⁡_ of 4.0 s. The CL intensities generated by online ultrasonically degassed solutions and general solutions were compared, and results showed that the CL intensity from the degassed solutions decreased obviously with regard to the CL intensity from general solutions, suggesting the oxygen dissolved in solution with solubility of 0.26 mM [40] was involved in the CL reaction. Furthermore, it is known that Co^2+^ can catalyze luminol-dissolved oxygen CL reaction, with O_2_^−·^ being increased which led to the CL intensity from luminol being remarkably enhanced [41]. While in the presence of UA, O_2_^−·^ would react with UA and oxidize UA to allantoin [42]. Thus the quenching mechanism of UA on luminol-Co^2+^ CL reaction can be explained as the decreased amount of O_2_^−·^, which was consumed by UA oxidation reaction, causing the CL intensity of luminol-Co^2+^ system to be inhibited. 

## 4. Applications

### 4.1. Real-Time Monitoring of UA Excretion in Human 24 h Urine

**Figure 5:**
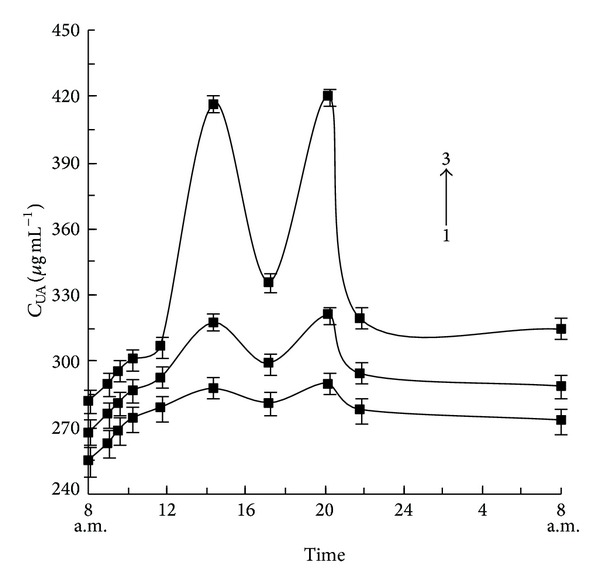
Metabolism of UA in human urine with different food during 24 h. Curve 1: porridge; Curve 2: vegetables; Curve 3: meats.

Three volunteers refrained from vegetables and meats intake for 1 day prior to the experiment. In the following 3 consecutive days, volunteers ingested diets at 12:30 am and 6:30 pm daily, with porridge, vegetables, and meats, respectively. On the 3rd day, the urine samples were periodically collected from volunteers and detected by the proposed CL method, with results being summarized in Table 2. It can be seen that the total UA excreted in 24 h urine from the three volunteers were 741.6, 797.7, and 878.4 mg, respectively, which indicated that the UA excretions were related to food intake with highest UA for meat intake and lowest UA for porridge intake. The details of UA metabolism in urine during 24 h were given in Figure 5, showing that the concentrations of UA simultaneously reached the maximum at 2 h after meals with the values of 288, 318, and 417 *μ*g mL^−1^, corresponding to porridge, vegetables, and meats intake, respectively.

### 4.2. Determination of UA in Human Serum Samples

The proposed method was also applied to determining UA in samples of human serum. The serum samples were collected from two volunteers, with no. 1–3 from volunteer 1 and no. 4–6 from volunteer 2. It was found that the serum UA concentrations were 38.0 ± 1.5–51.4 ± 0.6 *μ*g mL^−1^, which were in the normal range of 22–77 *μ*g mL^−1^ [43]. To validate the proposed method for UA determination, recovery studies were carried out on samples to which known amounts of UA (5.0, 7.0, and 10.0 ng mL^−1^) were added. The results were listed in Table 3 with recoveries varied from 96.6% to 106.4% and RSDs less than 3.5%, which confirmed that this presented FI-CL method was capable of UA quantification.

## 5. Conclusion

A rapid and cost-effective FI-CL method with luminol-Co^2+^ system for quantitative monitoring of urine UA excretion in 24 h at nanomolar level was described for the first time. In comparison with other reported methods for determining UA (Table 4), this proposed FI-CL analysis was attractive in relatively high sensitivity and wide linear range. Despite the fact that the sensitivity was a little inferior to the previous static CL for UA determination [32], the current FI-CL approach provided an amazing ST of 100 h^−1^, confirming the promise of this method for quantitative monitoring and real-time UA analysis.

## Figures and Tables

**Table 1:** Stability and reproducibility test of the FI-CL system for UA determination^a^.

Time (day)	*I*_0_ (blank)	RSD (%)	*I*_*s*_ 10.0 (nmol L^−1^)	RSD (%)	*I*_*s*_ (100.0 nmol L^−1^)	RSD (%)
1st	943	2.8	658	2.6	421	2.2
2nd	941	2.9	660	2.5	417	2.4
3rd	938	3.1	656	2.7	415	2.5
4th	945	2.7	662	2.5	420	2.3
5th	934	3.2	654	2.8	414	2.6

^a^The average of five determinations.

**Table 2:** Determination of 24 h urine UA excretion^a^.

Volunteers no.	Diets	*m* ^b^/*v* ^c^ (mg/mL)	*C*_UA_^d^ (*μ*g mL^−1^)
1	Porridge	741.6 ± 20.0/2700	274.7 ± 7.4
2	Vegetables	797.7 ± 25.6/2730	292.2 ± 9.4
3	Meats	878.4 ± 27.2/2670	329.0 ± 10.2

^a^The average of five determinations; ^b^the content of UA in urine sample; ^c^the volume of urine; ^d^the concentration of UA, calculated by b/c.

**Table 3:** Determination for UA in human serum samples^a^.

	Samples no.	Added/Found (ng mL^−1^)	RSD (%)	Recovery (%)	*C*_UA_^b^ (*μ*g mL^−1^)
Volunteer 1	1	0/7.6	3.3	96.6	38.0 ± 1.5
		5.0/12.4	2.4		
	2	0/8.1	3.0	103.1	40.2 ± 1.2
		10.0/18.4	1.7		
	3	0/7.9	3.1	97.8	39.5 ± 1.4
		7.0/14.7	2.1		

Volunteer 2	4	0/9.8	2.8	104.3	49.1 ± 0.8
		5.0/15.0	2.0		
	5	0/10.1	2.7	105.7	50.5 ± 0.7
		7.0/17.5	1.9		
	6	0/10.3	2.6	106.4	51.4 ± 0.6
		10.0/20.9	1.6		

^a^The average of five determinations; ^b^the average concentration of UA (*n* = 5).

**Table 4:** Comparison of different methods for UA determination.

Methods	Linear ranges (*μ*mol L^−1^)	LODs (*μ*mol L^−1^)	Samples	References
CE	10~5.0 × 10^2^	3.3	Human urine and serum	[7]
EA	10~1.6 × 10^2^	2.6	Human serum	[8]
HPLC	5~1.0 × 10^3^	1.5	Human serum	[9]
	10~5.0 × 10^2^	4.3 × 10^−1^	Human serum	[10]
DPV	1~1.6 × 10^2^	1.9 × 10^−1^	Human urine and serum	[11]
	3.0 × 10^−1^~1.0 × 10^2^	3.0 × 10^−1^	Human urine	[12]
UV-Vis	1~1.0 × 10^2^	3.0 × 10^−1^	Human urine	[13]
	10~1.0 × 10^2^	3.0	Human urine	[14]
CL	1.0 × 10^−4^~7	1.1 × 10^−5^	Human urine and serum	[32]
FI-CL	4.8~1.7 × 10^2^	2.9	Human urine and serum	[15]
	4.0 × 10^−1^~2.0 × 10^2^	1.2 × 10^−1^	Human serum	[16]
	3.0 × 10^−2^~3	1.0 × 10^−2^	Human urine and serum	[17]
	3.0 × 10^−3^~5.0 × 10^−1^	1.0 × 10^−3^	Human urine and serum	This study
